# Dendritic/Post-synaptic Tau and Early Pathology of Alzheimer’s Disease

**DOI:** 10.3389/fnmol.2021.671779

**Published:** 2021-06-25

**Authors:** Xiaomin Yin, Chenhao Zhao, Yanyan Qiu, Zheng Zhou, Junze Bao, Wei Qian

**Affiliations:** ^1^Department of Biochemistry and Molecular Biology, Medical School, Nantong University, Nantong, China; ^2^Jiangsu Key Laboratory of Neuroregeneration of Jiangsu and Ministry of Education of China, Co-innovation Center of Neuroregeneration, Nantong University, Nantong, China; ^3^NMPA Key Laboratory for Research and Evaluation of Tissue Engineering Technology Products, Nantong University, Nantong, China

**Keywords:** tau, Alzheimer’s disease, cognitive impairment, post-synapse, synaptic localization

## Abstract

Microtubule-associated protein tau forms insoluble neurofibrillary tangles (NFTs), which is one of the major histopathological hallmarks of Alzheimer’s disease (AD). Many studies have demonstrated that tau causes early functional deficits prior to the formation of neurofibrillary aggregates. The redistribution of tau from axons to the somatodendritic compartment of neurons and dendritic spines causes synaptic impairment, and then leads to the loss of synaptic contacts that correlates better with cognitive deficits than amyloid-β (Aβ) aggregates do in AD patients. In this review, we discuss the underlying mechanisms by which tau is mislocalized to dendritic spines and contributes to synaptic dysfunction in AD. We also discuss the synergistic effects of tau and oligomeric forms of Aβ on promoting synaptic dysfunction in AD.

## Introduction

Tau is a microtubule-associated protein (MAP) that participates in microtubule assembly and stabilization (Weingarten et al., 1975; Drechsel et al., 1992). Tau protein has a very dynamic interaction with microtubules (Konzack et al., 2007). This highly dynamic interaction had been described as a kiss and hop mechanism with 40 ms dwell time (Igaev et al., 2014; Janning et al., 2014).

Tau is encoded by *MAPT* gene including 16 exons on chromosome 17q21 (Neve et al., 1986). Six molecular isoforms of tau are generated from alternative splicing of exons 2, 3, and 10 in *MAPT* gene transcripts in human brain (Amos, 2014). These six tau isoforms including three 3R taus (0N3R, 1N3R, and 2N3R) and three 4R taus (0N4R, 1N4R, and 2N4R), differ in containing three (3R taus) or four (4R taus) microtubule binding repeats (R) of 31–32 amino acids in the carboxyl terminal half and zero (0N), one (1N), or two (2N) amino terminal inserts of 29 amino acids each (Iqbal et al., 2009). Tau is phosphorylated at serine/threonine sites or/and tyrosine sites, and the biological activity of tau is regulated by the degree of phosphorylation modification (Lindwall and Cole, 1984; Kopke et al., 1993; Alonso et al., 1994). All of the six tau isoforms are hyperphosphorylated and aggregated into neurofibrillary tangles (NFTs) in Alzheimer’s disease (AD) brain (Grundke-Iqbal et al., 1986; Iqbal et al., 1986, 1989; Lee et al., 1991; Goedert et al., 1992). A disease-like pseudohyperphosphorylation of tau dramatically diminishes the tau–microtubules interaction (Niewidok et al., 2016). Interestingly, tau protein in the brain becomes highly phosphorylated during hibernation in different species, such as arctic ground squirrel, Syrian hamsters and black bears, and this hyperphosphorylation is reversed after arousal (Su et al., 2008; Stieler et al., 2011). To date, the majority research of tau pathology has been concentrated on the processes of tau aggregation and its subsequent toxicity to neurons. In this review, we focus on the mislocalization of tau from axons to dendrites and post-synapses. It is an earlier event in AD pathogenesis prior to the formation of amyloid plaques and NFTs and therefore may be a preferable therapeutic target.

## Synaptic Localization of Tau

### Tau Distribution Under Physiological Conditions

**FIGURE 1:**
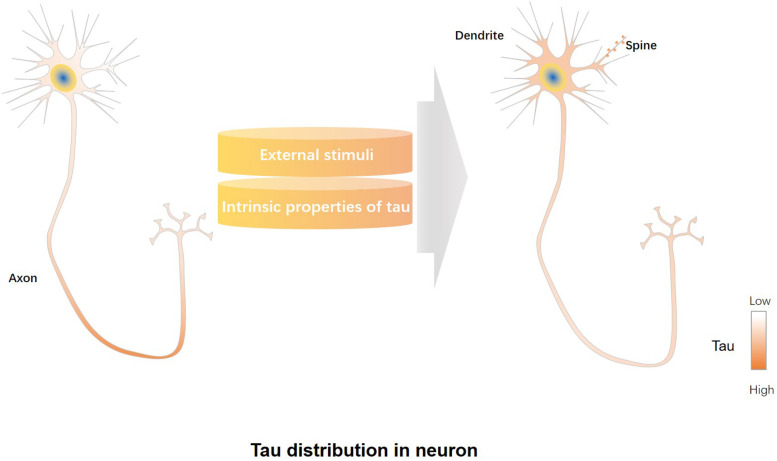
Regulatory factors involved in mislocalization of tau to dendrites/post-synapses. Under physiological conditions, most of tau is localized in the distal segment of the axon, whereas lower tau concentrations are found in the proximal segment of the axon, and the lowest levels in the soma and dendrites **(left panel)**. External stimuli, including increased neuronal activity or treatment with amyloid-beta (Aβ) or glucocorticoids, can promote dendrite/post-synaptic localization of tau, while intrinsic properties of tau, such as those caused by altered tau splicing, truncations, phosphorylation and acetylation modifications, affect the dendrite/post-synaptic translocation of tau **(middle panel)**. Tau redistributes to the somatodendritic compartment and post-synapses under pathological conditions **(right panel)**.

Most of tau localizes in the distal segment of the axon, lower concentrations are found in the proximal segment of the axon, and the lowest levels in the soma and dendrites (Black et al., 1996; Mandell and Banker, 1996) (Figure 1). It is reported that approximately three times more tau exists in the white matter (mainly containing axons) than the gray matter (mainly containing dendrites and cell bodies) of rat and bovine brains (Binder et al., 1985). Studies also found endogenous tau localized in dendrites and post-synapses of rodent neurons (Ittner et al., 2010; Mondragon-Rodriguez et al., 2012b; Zempel et al., 2013; Kimura et al., 2014; Swanson et al., 2017). Endogenous murine tau was shown to localize to dendritic spines (Xia et al., 2016). Furthermore, physiological tau was observed both at presynaptic and postsynaptic termini in human brains (Tai et al., 2012). The post-synaptic localization of tau suggests that tau has physiological functions not only in the axon but also in the synapse (Ittner et al., 2010; Kimura et al., 2014; Regan et al., 2015). Tau plays roles in microtubule stabilization and axonal transport as an axonal cytoskeletal protein (Guo et al., 2017), whereas synaptic tau engages in neuronal signaling and synaptic plasticity (Ittner et al., 2010; Chen et al., 2012).

### Possible Mechanisms of Physiological Tau Distribution

The underlying mechanisms responsible for the axonal tau gradient are in dispute. Evidence suggests several overlapping mechanisms. First, since the affinity of tau to axonal microtubules is higher than that of dendrites or soma in neurons, tau is predominantly localized to axons (Kanai and Hirokawa, 1995; Hirokawa et al., 1996). The axon initial segment (AIS) also provides a barrier limiting the amount of tau to relocate from axons to the soma (Li et al., 2011). This retrograde diffusional restriction of tau may be governed by several protein-protein interactions of tau with AIS complexes (such as ankyrin G/EB1, GSK3β) (Zempel et al., 2017). Second, tau is selectively degraded in the somatodendritic compartment by autophagy and proteasomes. Third, the distribution of tau mRNA connects with native introns and untranslated regions UTR (Kanai and Hirokawa, 1995; Hoover et al., 2010).

## Tau Binding Proteins in the Post-synapse

**FIGURE 2:**
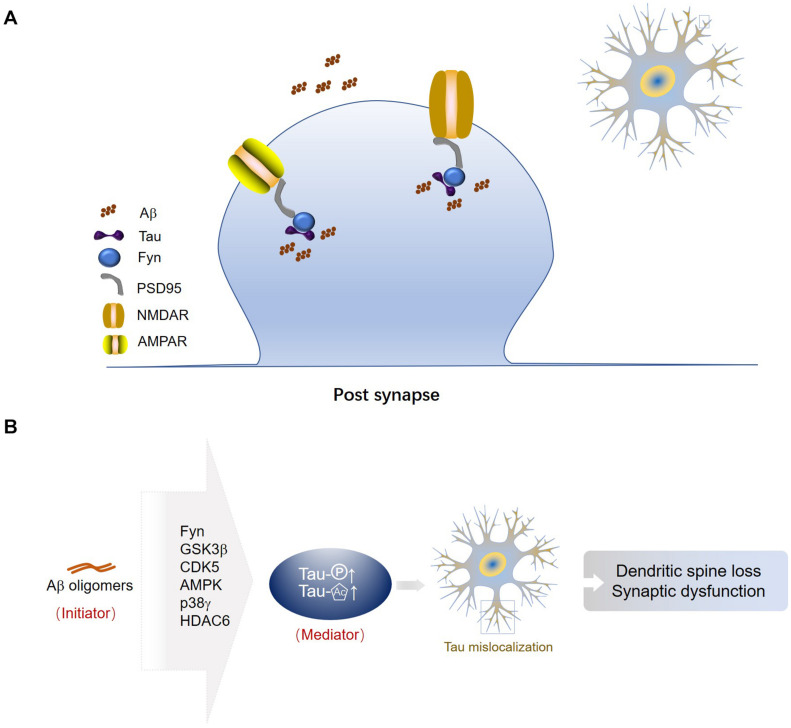
Tau associated proteins in post-synapses. **(A)** Tau-Fyn-PSD95-post-synaptic receptor complex in the post-synaptic compartment. Tau, Fyn, PSD-95, and NMDARs or AMPARs are predicted to form a protein complex at the synapse. **(B)** Aβ oligomers are an initiator of synaptic dysfunction, and synaptic tau seems to be an indispensable mediator in the progress. Fyn is the mediator between Aβ and tau. GSK3β may be a central factor linking extracellular Aβ and intracellular tau. Aβ may activate CDK5 to phosphorylate tau at synaptic sites. AMPK and p38γ may take part in tau phosphorylation induced by Aβ, but subsequently they have opposite effects on synaptic function. Aβ inhibits activity of HDAC6 and increases acetylation levels of tau, resulting in loss of tau polarization.

Tau interacts with the Src family kinase Fyn *via* its Proline-X-X-Proline (PXXP) motifs in the Proline-rich region (Lee et al., 1998; Ittner et al., 2010; Lau et al., 2016). Fyn fails to localize to the synapse in tau-depleted neurons (Ittner et al., 2010; Sapir et al., 2012). The tau/Fyn complex binds to PSD-95 (post-synaptic density protein 95) at the post-synapse (Ittner et al., 2010; Mondragon-Rodriguez et al., 2012b; Lopes et al., 2016). PSD-95 is a key scaffolding protein for post-synaptic receptors (Kim and Sheng, 2004). The interaction between PSD-95 and NMDAR or AMPAR is primarily mediated by the postsynaptic density-95, disks-large, zona occludens 1 (PDZ) domains (Christensen et al., 2019). Tau connects with PSD-95 as well as NMDARs or AMPARs, the post-synaptic receptors (Ittner and Ittner, 2018). Tau, Fyn, PSD-95, and NMDARs are predicted to form a protein complex at the synapse (Mondragon-Rodriguez et al., 2012b) (Figure 2A).

The localized activity of Fyn is critical for NMDAR-mediated excitotoxicity (Ittner et al., 2010; Knox et al., 2014; Miyamoto et al., 2017). Fyn phosphorylates the subunit 2B (NR2B) of NMDAR at tyrosine-1472 of cytosolic C-terminus, which promotes the interaction between NR2B and PSD-95 and results in toxic downstream signaling pathways in neurons (Rong et al., 2001). Decreased synaptic clustering of both NMDA and AMPA receptors is induced by the infiltration of tau into dendritic spines, thereby resulting in compromised excitatory synaptic transmission and memory deficits (Hoover et al., 2010; Xia et al., 2015; Kim et al., 2016; Yin et al., 2016; Zhao et al., 2016).

Tau interacts with microtubules and regulates their turnover to modulate the dynamic movement of microtubules into and out of dendritic spines, which is important to long-term depression (LTD) induction (Kapitein et al., 2011). Similarly, tau influences the cross-talk between microtubules and actin cytoskeletal networks through direct binding to actin (Fulga et al., 2007) or regulation of microtubule entry into spines (Jaworski et al., 2009), which is an important regulator of synaptic function and AMPAR trafficking (Zhou et al., 2001, 2004).

## Role of Tau in Post-synapse

Tau in post-synapse is crucial for LTD (Kimura et al., 2014). Transfection of tau shRNA prevented the induction of LTD but not LTP. The necessary role of tau in the hippocampal LTD is supported by the rescued LTD phenotype when endogenous tau was replaced with human tau. The requirement for tau in hippocampal LTD is also verified by the selective deficits in spatial reversal learning observed in tau knockout mice (Regan et al., 2015).

Tau also appears to modulate LTP since exposure of neurons to tau inhibits hippocampal LTP in rat hippocampal synapses (Ondrejcak et al., 2018). Similarly, the LTP inhibition is dependent on tau because co-injection with an antibody against tau can prevent the LTP inhibition caused by extracts of Alzheimer’s disease brain (Ondrejcak et al., 2018).

It has been identified that tau plays an important physiological role in synapses by studies using tau knockout mice (Ke et al., 2012; Ahmed et al., 2014; Biundo et al., 2018). Further investigations are still required to detect the physiological roles of tau in synapses. Tau acts as a mediator of AD-related synaptic deficits, which has been demonstrated by numerous studies (Spires-Jones and Hyman, 2014). The increased concentration of tau in dendrites may affected memory and synaptic plasticity. Mislocalized tau reduces the miniature excitatory postsynaptic currents (mEPSCs) in rats. A silencing of synapses and reduction of AMPARs in the post synaptic site accompany a decrease in mEPSCs (Hoover et al., 2010). Dendritic tau is mostly hyperphosphorylated (Brion et al., 1994; Gotz et al., 1995; Tashiro et al., 1997; Ittner et al., 2010; Shentu et al., 2018), dissociated from microtubules and related to dendritic spine loss (Thies and Mandelkow, 2007). Dendritic tau is involved in dendritic loss, aberrant post-synaptic activity and cognitive dysfunction in AD or other tauopathies (Hoover et al., 2010; Zhao et al., 2016; Bories et al., 2017). Overexpression of tau in cultured neurons and AD mice increases tau in the somatodendritic compartment (Zempel et al., 2017). Importantly, the increased postsynaptic tau was linked to spine loss in a tau transgenic mouse model (Zempel et al., 2017; Ittner and Ittner, 2018). Synaptic loss is the earliest indication of neuronal malfunction and the best biological correlate of disease progression in AD and related tauopathies (Masliah et al., 1991; Terry et al., 1991; Scheff et al., 2006). Under pathological conditions, such as in human tauopathy or animal models of tau overexpression, the presence of tau at synapses is more apparent, suggesting a role for tau in disease pathogenesis (Hoover et al., 2010; Ittner et al., 2010; Pooler et al., 2014; Sokolow et al., 2015; Dejanovic et al., 2018; Ji et al., 2019; Kubo et al., 2019).

## External Stimuli Leading to Post-synaptic Distribution of Tau

Neuronal activity and extracellular signals regulate dendritic/post-synaptic localization of tau. Increased neuronal activity or treatment with amyloid-beta (Aβ) or glucocorticoids can increase the dendritic and synaptic localization of tau (Frandemiche et al., 2014; Pinheiro et al., 2016). Long-term potentiation (LTP), a synaptic plasticity mechanism critical for memory formation (Bannerman et al., 2014), can increase post-synaptic levels of tau (Frandemiche et al., 2014). Engagement and redistribution of post-synaptic glutamate receptors is critical to synaptic plasticity (Paoletti et al., 2013). In cultured neurons, activation of post-synaptic glutamate receptors induces translocation of tau from dendritic shafts into post-synaptic densities (Ittner et al., 2010; Frandemiche et al., 2014). Glucocorticoid treatment leads tau localization to soma and dendrites (Sotiropoulos et al., 2011; Lopes et al., 2016). Exposure to oligomeric Aβ increases levels of tau in dendrites in AD (Zempel et al., 2010). In general, external stimuli regulate dendritic localization of tau (Figure 1).

## Intrinsic Properties of Tau Related to Post-synaptic Location

### Isoforms of Tau

Different tau isoforms may contribute to their dendritic and post-synaptic recruitment. 2N tau is prone to sorting into the somatodendritic compartment, compared to other tau isoforms (Zempel et al., 2017). Whether 3R and 4R tau isoforms are differentially localized to dendrites/post-synapses remains elusive (Ittner and Ittner, 2018).

### Truncations of Tau

Caspase-2 cleaves tau at Asp314 to generate △tau314, which is necessary for tau to mislocalize to dendritic spines (Zhao et al., 2016). In addition, the caspase-3 cleavage of tau at Asp421 may contribute to tau synaptic propagation in neurons (Kim et al., 2016; Nicholls et al., 2017). Several lines of evidence suggest that truncated forms of tau may also contribute to synaptic dysfunction. Mutating Asp314 on tau to prevent the caspase-2 cleavage of human P301L tau blocks mislocalization of tau to the dendritic compartment and prevents cognitive impairment in AD mouse models (Zhao et al., 2016). Normal memory and dendritic spine morphology are exhibited in J20 APP transgenic mice lacking caspase-2 (Pozueta et al., 2013).

### Post-translational Modifications of Tau

Tau undergoes several post-translational modifications (PTMs), including phosphorylation, acetylation, ubiquitination, methylation, and glycosylation (Morris et al., 2015).

Tau has more than 45 phosphorylation sites mainly located in its proline-rich domain and C-terminal domain (Mondragon-Rodriguez et al., 2008a,b, 2012a,b, 2014; Regan et al., 2015). Phosphorylation of tau directly affects the distribution of tau to dendrite and post-synapse. Individual phosphorylation-mimicking tau mutants at T231/S235, S262/S356, or S396/S404 enhances localization of tau into dendritic spines in cultured neurons (Xia et al., 2015). Phosphorylation-mimicking tau with 14 simultaneous mutant sites induces tau to dendritic spines in cultured neurons, then reduces AMPA receptors and finally leads to synaptic impairments (Hoover et al., 2010; Miller et al., 2014). Furthermore, increased tau phosphorylation causes mislocalization of tau to post-synaptic sites both in AD brain and in transgenic mice overexpressing P301S tau (Tai et al., 2012; Dejanovic et al., 2018). A series of studies indicate that one of the potential mechanisms responsible for post-synaptic localization of tau may be the association of tau with the tyrosine kinase Fyn. Increasing phosphorylation level of tau at sites such as Ser396, Ser404, Thr205, Thr231, and Ser235 promotes dissociation of the Tau/Fyn/PSD95 complex, which is crucial for LTD induction (Mondragon-Rodriguez et al., 2012a,b). In the same regard, phosphorylation of tau at Ser396 is necessary for the expression of hippocampal LTD (Regan et al., 2015). Non-phosphorylated tau contributes to LTP, while phosphorylated tau contributes to LTD (Shipton et al., 2011; Mondragon-Rodriguez et al., 2012a,b; Regan et al., 2015; Ittner et al., 2016). Tau phosphorylation, induced by the Pro301 to Leu301 mutation linked to FTDP-17, also weakens the AIS barrier by structurally modifying the AIS and shifting its location (Hatch et al., 2017).

Bovine tau is modified by *O*-GlcNAcylation, a unique type of *O*-glycosylation for cytosolic proteins (Arnold et al., 1996). Human brain tau is also *O*-GlcNAcylated, and *O*-GlcNAcylation regulates phosphorylation of tau in a site-specific manner (Liu et al., 2004). Reduced *O*-GlcNAcylation of tau results in hyperphosphorylation of tau (Gong et al., 2006; Deng et al., 2009). *O*-GlcNAcylation of tau prevents tau from oligomerization and decreases neuronal cell loss (Yuzwa et al., 2012).

Acetylation of tau in post-synapse participates in an activity-dependent pathway regulating synaptic plasticity and memory. Acetylation-mimicking tau mutants reduce Kidney/Brain (KIBRA) protein, a known regulator of AMPARs and memory (Makuch et al., 2011), and AMPAR presentation, and impairs hippocampal LTP (Tracy et al., 2016). This impairment affects memory and is associated with AD (Forner et al., 2017). Acetylated tau reduces KIBRA and impairs LTP by impeding activity-induced actin polymerization and thereby affecting postsynaptic membrane localization of AMPA receptors, which implicates the vital function of tau in regulating synaptic plasticity (Tracy and Gan, 2017). Acetylation of tau destabilizes the AIS, weakens the barrier, and allows retrograde redistribution of tau into the somatodendrites (Sohn et al., 2016).

## Tau and Aβ

Amyloid-β may initiate mislocalization of tau at dendrites, which in turn can affect accumulation of Aβ. Oligomerized Aβ can only increase mislocalization of tau to the dendrites when tau is phosphorylated (Miller et al., 2014). Fyn is the mediator between Aβ and tau. Aβ promotes the post-synaptic localization of tau, and then the increased concentration of tau attracts Fyn to phosphorylate and activate NMDA receptor and triggers excitotoxicity due to calcium dyshomeostasis (Ittner et al., 2010). It has been reported that decreasing tau levels in Aβ-forming AD mouse models prevented the post-synaptic dysfunction (Roberson et al., 2007, 2011; Ittner et al., 2010; Bi et al., 2017). In contrast, increasing tau levels in amyloidosis mouse models enhanced synaptic loss and memory impairment (Ittner et al., 2010; Chabrier et al., 2014). Tau phosphorylation contributes to dendritic spine loss (Mairet-Coello et al., 2013) and neuronal death (Tackenberg et al., 2013) induced by Aβ. Colocalization of Aβ and tau is shown in approximately one third of synapses in AD brain (Fein et al., 2008) (Figure 2A). GSK3β may be the central factor linking extracellular Aβ and intracellular tau (Mandelkow, 1999; Dewachter et al., 2009), since Aβ can increase phosphorylated tau while GSK3β inhibition can block the increasing of phosphorylated tau and prevent Aβ-induced impairment of LTP in mice (Shipton et al., 2011). Deregulation of Cdk5 caused by the accumulation of p25, a truncated fragment of p35, contributes to the pathogenesis of AD. The p25/Cdk5 kinase phosphorylates tau efficiently and hinders tau from binding to microtubules (Patrick et al., 1999). Proteomics data suggest that misfolded Aβ may activate CDK5 to phosphorylate tau at synaptic sites (Wu et al., 2018). Eliminating the specific AMPK phosphorylation of tau prevents the Aβ-induced loss of dendritic spines and restores synaptic functions (Mairet-Coello et al., 2013; Zempel et al., 2013). On the contrary, post-synaptic p38γ-mediated tau phosphorylation alleviates Aβ-induced excitotoxicity, suggesting the protective function of tau phosphorylation in the post-synaptic compartment (Ittner et al., 2016). Oligomeric Aβ was shown to inhibit activity of histone deacetylase 6 (HDAC6) and increase acetylation levels of tau, resulting in loss of tau polarization (Tsushima et al., 2015).

## Conclusion

In the present review, we discuss somatodendritic and post-synaptic localizations of tau under both physiological and pathological conditions. Mislocalization of tau to dendrites and post-synapses triggered by external and intrinsic factors is an early event in AD pathogenesis before tau aggregation (Figure 1). Synaptic tau, a mediator of AD-related synaptic deficits, associates with the onset of cognitive decline in AD. Synaptic impairments take place much earlier in AD pathogenesis than the formation of NFTs. Synaptic loss is highly correlated with cognitive decline and is the first indicator of AD progress. Abnormal PTMs of tau, such as phosphorylation, acetylation, and truncation, can all contribute to its mislocalization at synaptic sites. Aβ and tau drive synaptic dysfunction synergistically, which is the latent initial crisis in AD (Figure 2B). Therefore, therapeutic strategies targeting synaptic tau might be promising in intervening early pathological events in AD.

## Author Contributions

WQ wrote the draft of the manuscript. All authors contributed to the manuscript revisions, as well as read and approved the submitted version.

## Conflict of Interest

The authors declare that the research was conducted in the absence of any commercial or financial relationships that could be construed as a potential conflict of interest.
